# Animals, quality and the pursuit of relevance

**DOI:** 10.1242/dmm.049775

**Published:** 2022-10-17

**Authors:** Karen L. Svenson, Stephen D. Krasinski, Michael Ellis, Nadia Rosenthal, Edison T. Liu, Kenneth H. Fasman

**Affiliations:** The Jackson Laboratory, Bar Harbor, ME 04609, USA

## Abstract

In 2021, the National Institutes of Health Advisory Committee to the Director (ACD) announced recommendations to improve the reproducibility of biomedical research using animals. In response, The Jackson Laboratory faculty and institutional leaders identified key strategies to further address this important issue. Taking inspiration from the evolution of clinical trials over recent decades in response to similar challenges, we identified opportunities for improvement, including establishment of common standards, use of genetically diverse populations, requirement for robust study design with appropriate statistical methods, and improvement in public databases to facilitate meta-analyses. In this Perspective, we share our response to ACD recommendations, with a specific focus on mouse models, with the aim of promoting continued active dialogue among researchers, using any animal system, worldwide. Such discussion will help to inform the biomedical community about these recommendations and further support their much-needed implementation.

## Teaser

**Summary:** Lack of reproducibility in animal studies continues to be a challenge, and improving this is a National Institutes of Health priority. Key strategies to enhance translatability are offered and discussed by The Jackson Laboratory investigators.

## Introduction

Recognizing the need to restructure preclinical studies using animals to improve reproducibility and translatability, the biomedical research community continues to improve mammalian models of human disease and their utility in research. The National Institutes of Health (NIH) has made this a high priority (Collins and Tabak, 2014), recently creating a working group of the Advisory Committee to the Director (ACD) charged with making recommendations for enhanced rigor, translatability and transparency of such studies. The report from the working group was made public in the summer of 2021 (https://acd.od.nih.gov/working-groups/eprar.html) and contains recommendations focused on improving practices outlined in five themes: (1) improve study design and data analysis; (2) identify questionable practices, including incomplete reporting; (3) explicitly define the selection and relevance of animal models used; (4) expand methodological documentation in terms of environmental confounders (e.g. bedding, microbiome) and the long-term care of large animals; and (5) evaluate the cost and effectiveness of implementing these sweeping recommendations.

We wholeheartedly concur with the recommendations submitted by the working group, and herein offer additional comments and recommendations that may further refine the implementation of the report. Our comments are focused on the mouse because it is the most widely used mammalian species in biomedical research, and thus improvements in this sector of animal experimentation will have a very wide impact. Although the implementation specifics will vary with differing model systems, the overall recommendations remain relevant across all preclinical model systems.

The Jackson Laboratory (JAX) has been a key player in the field of mammalian genetics research and is a major provider of mouse models and services for the research community. The issues discussed in the themes addressed by the working group are at the heart of research and resource generation at JAX and core to our mission as a not-for-profit institution. Our perspective, however, is extensible to the global community of biomedical investigators using animals to improve human health.

## Timely deposition of animal models and their validation data into public repositories

Currently, investigators cannot publish a data-intensive paper without depositing the data in a public data repository, but they can publish a paper describing a new mouse model without depositing the strain into an animal repository. Some investigators delay submission to a public repository, retaining their models for multiple studies over many years before making them publicly available. Because the best validation of a model is recapitulation of the published phenotype by another laboratory, delayed submission to a repository is an impediment to enforcing reproducibility. Further, if a model cannot be validated, these negative results are not published or recorded, resulting in a significant waste of time and resources as others can benefit from the knowledge of such negative results. We recommend that the Resource Sharing Plan in a grant application includes a commitment to timely deposition of a validated animal model into a suitable public repository. Although this may lead to increased cost and time to recover resources from such repositories, the cost is far below that of expanded experimentation with poorly validated and unstable models. Individual laboratories are not usually prepared to provide or receive live animals due to health restrictions of importation and the infrastructural costs for distribution. Therefore, additional resources and centralized centers will be needed to expand and distribute quality-controlled models. We further recommend that guidelines for model validation are established and that a suitable depository for submitting negative validation data is instituted. A framework for such a database exists in the Mouse Genome Informatics (http://www.informatics.jax.org) resource. Ultimately, it may be more efficient to empower an animal model repository to not only hold the physical model, but also all the associated validation data. When models are deposited in a repository, details are explicit and shared. If the repository is sufficiently funded, it can serve as an independent third party, providing characterization, validation and documentation. Moreover, using cryopreservation techniques, repositories can mitigate genetic drift (Donahue et al., 2012). For the mouse, the Mutant Mouse Resource and Research Centers (MMRC) act to an extent to fulfill this function (https://www.mmrrc.org/).

## The importance of genetic background

The original purpose for developing inbred mice was to observe experimental effects on a controlled, homogeneous genetic background. However, small changes in the genetic background from genetic drift due to prolonged isolated propagation of a reference stock have often confounded reproducibility (Stevens et al., 2007). All reference stocks need to have their genomes sequenced and laboratories may need to ensure that their ‘in-house’ reference strains have not undergone genetic drift. It is also known that a genetic lesion can manifest very different phenotypes in varying strain backgrounds (Taft et al., 2006). Indeed, by transferring a particular lesion onto a variety of inbred backgrounds, important genetic modifiers of disease outcome have been identified (Ouellette et al., 2020). Further, deliberate generation of a genetically diverse mouse resource population can be the basis for more precise models of disease and enhance the relevance of mouse models to human populations. JAX, along with the University of North Carolina and Tel Aviv University, created the genetically diverse Collaborative Cross and Diversity Outbred mouse resources (Churchill et al., 2012), encompassing up to 90% of the population variability found in inbred mice. By utilizing a genetically diverse panel of inbred strains to study the effects of a precise mutation, we mimic the diversity of human genetics in disease traits, an achievement that cannot be accomplished by using only a single commonly used strain, such as C57BL/6. This critical consideration of genetic background has also driven the development of the Hybrid Mouse Diversity Panel (Ghazalpour et al., 2012), a large collection of well-characterized inbred mouse strains designed to facilitate analysis of complex traits. We recommend supporting research into new strategies that capture genetic variation of human populations using genetically diverse mouse panels. This is also especially relevant to the genetic background used to introduce specific CRISPR mutations.

“Many of the elements recommended by the NIH working group have parallels in the evolution of the design and conduct of clinical trials.”

## Centers of excellence to promote best practices, and economies of scale and training

**Fig. 1.**
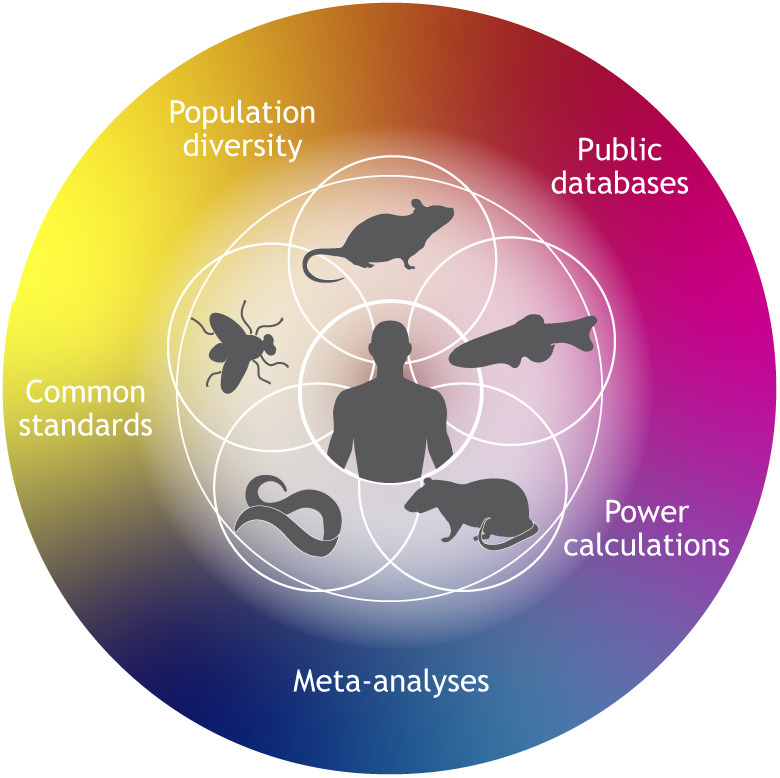
**A diversity of animal systems has enabled important advances in modeling human disease.** Key strategies for improving translation of these models include establishment of common research standards, use of diverse genetic backgrounds within a species, requiring adequate statistical power and better access to public databases to facilitate meta-analyses of multi-omics data.

All elements of biological experimentation face challenges of rigor and reproducibility and often the solution has been a formal enunciation of the criteria for rigorous design and imposition of statistical formalism (Gamble et al., 2017). The most pertinent transition has been the conduct of human clinical trials over the past 70 years, where statistical rigor is a fundamental requirement (Homer et al., 2022). Requiring appropriate statistical power and data quality control was a significant challenge early in the evolution of human clinical trials. This was resolved by developing larger cooperative clinical trials groups, because no single institution could mount a study with sufficient power. Translation of preclinical studies using animals will be improved by similar requirements in study design, including appropriate power calculations to estimate sample and cohort sizes, use of genetically diverse subjects, use of consistent and commonly accepted standards for measuring outcome and public access to data for additional analysis (Fig. 1). Many of the elements recommended by the NIH working group have parallels in the evolution of the design and conduct of clinical trials. What is lacking in current preclinical experimentation today has been previously overcome in clinical trials:

- **Power calculations.** Too often preclinical studies lack power calculations while focusing on minimizing costs. In human clinical trials, there is a formalism in the statistical analysis and the use of results in phase I-III clinical studies of different cohort sizes (https://www.fda.gov/regulatory-information/search-fda-guidance-documents/statistical-guidance-reporting-results-studies-evaluating-diagnostic-tests-guidance-industry-and-fda; https://www.fda.gov/patients/drug-development-process/step-3-clinical-research#Clinical_Research_Phase_Studies). No such formalism exists for preclinical studies, although resources such as The Experimental Design Assistant have recently been developed to address this issue (Percie du Sert et al., 2017).
- **Population diversity.** Clinical trials often seek to balance consistency and diversity. Earlier, it was not surprising to see a study that recruited only 50- to 70-year-old Caucasian men and to have these results generalized to all human populations. The problem in preclinical studies is more acute when experimentation is restricted to only one inbred line.
- **Common standards.** The clinical trials community spent significant effort defining response criteria. We are finding a lack of consistent standards in patient-derived xenograft and immuno-oncology treatment models even in calling response in the preclinical setting (Hafner et al., 2016; Guillen et al., 2022). For example, does one follow the clinical Response Evaluation Criteria in Solid Tumors (RECIST) criteria or alternative measurements such as modified RECIST (mRECIST) (Gao et al., 2015).
- **Databases and meta-analysis.** In clinical trials, all results, even unpublished, are in databases. Although there remains a challenge in integrating legacy databases and data access, at least they exist and can be leveraged. Establishing a national database, or a network of data centers where raw data of preclinical studies are published, should be considered. With such a database infrastructure, meta-analyses of preclinical studies can be performed. This also helps immensely with establishing standards.

We propose a parallel development in the preclinical space by forming Centers of Excellence (CoEs), structured to provide suitable scale for critical preclinical validation studies. Reproducible phenotyping requires careful attention to factors including instrumentation, staff training and protocol standardization across laboratories. A ‘center of excellence’ approach can offer a high-quality, cost-efficient and accessible way to provide these capabilities for researchers who would otherwise not be able to execute these studies in their individual laboratories. CoEs are an efficient means of maintaining expensive animal model colonies, and the vivarium requirements for large-scale and/or long-term experiments with special phenotyping needs, which is especially relevant for neurodegeneration or other aging studies.

Modeled after national facilities in high-energy physics and astronomy, such CoEs can accept preclinical proposals that require hundreds to thousands of mice – preferably across diverse strain backgrounds. Exploratory work can still be performed at individual institutions and laboratories. However, for robust and reproducible *in vivo* experiments, particularly for pivotal preclinical studies, the total funding for both the CoEs and experiments conducted at those centers may well be more cost effective than a large number of inadequately powered studies.

## Cost

We laud the ACD working group for highlighting cost considerations. We believe that our additional recommendations regarding model validation, deposition to repositories, understanding genetic background and investing in CoEs can result in more effective and efficient use of mouse models in animal experimentation, but are also cognizant of the cost of such a transition. Such changes are, however, absolutely necessary and may require larger budgets. Therefore, we encourage the NIH to assemble experts knowledgeable in the true costs of animal production and experimentation to work with experts in finance to construct cost models so we can collectively explore the most efficient and cost-effective means to achieve our common goals.

“From the outset, experiments reviewed in funding proposals must be highly scrutinized for key elements of study design that promote scientific rigor and clear a path to transparency and translatability.”

## Conclusions

Rigor in science drives advancement in medicine. From the outset, experiments reviewed in funding proposals must be highly scrutinized for key elements of study design that promote scientific rigor and clear a path to transparency and translatability. Recent successes in gene therapy, for example, have come about after rigorous preclinical experimentation often from multiple animal models (Doshi and Arruda, 2018). For any animal system used, clear identification of the model, its proper nomenclature and genetic background is essential to this path.

Biomedical research must be done responsibly, ethically and humanely, with full accountability. We applaud the efforts of the NIH to improve research involving animals, and, at JAX, we are committed to supporting integrative strategies to promote these efforts.
